# EZH2 Dysregulation and Its Oncogenic Role in Human Cancers

**DOI:** 10.3390/cancers17193111

**Published:** 2025-09-24

**Authors:** Shiv Verma, Nikita Goyal, Suhani Goyal, Parminder Kaur, Sanjay Gupta

**Affiliations:** 1 Department of Urology, School of Medicine, Case Western Reserve University, Cleveland, OH 44106, USA; sxv304@case.edu (S.V.); nikitagoyal2026@gmail.com (N.G.); suhanigoyal45@gmail.com (S.G.); pxk502@case.edu (P.K.); 2 The Urology Institute, University Hospitals Cleveland Medical Center, Cleveland, OH 44106, USA; 3 The Peddie School, 201 South Main Street, Hightstown, NJ 08520, USA; 4 Department of Pathology, Case Western Reserve University, Cleveland, OH 44106, USA; 5 Department of Pharmacology, Case Western Reserve University, Cleveland, OH 44106, USA; 6 Department of Nutrition, Case Western Reserve University, Cleveland, OH 44106, USA; 7 Division of General Medical Sciences, Case Comprehensive Cancer Center, Cleveland, OH 44106, USA

**Keywords:** biomarkers, enhancer of zeste homolog 2, polycomb repressive complex 2, oncogene, human cancer

## Abstract

Enhancer of Zeste Homolog 2 (EZH2) is a key epigenetic regulator known for its role in global gene silencing and is involved in a variety of cellular processes, including cell survival, proliferation, invasion, and self-renewal. As a core component of the Polycomb Repressive Complex 2 (PRC2), EZH2 catalyzes the trimethylation of histone H3 at lysine 27 (H3K27me3), leading to chromatin compaction and transcriptional repression. Dysregulated EZH2 expression is observed in a wide range of solid tumors and hematological malignancies and is frequently associated with increased metastatic potential and poor clinical outcomes. While EZH2 primarily mediates gene silencing through its canonical PRC2-dependent activity, it also exerts oncogenic effects via non-canonical mechanisms. In its non-canonical role, EZH2 acts independently of PRC2, interacting with other signaling molecules as a transcriptional activator or co-activator, thereby promoting the activation of oncogenic pathways. Through both canonical and non-canonical mechanisms, EZH2 significantly contributes to tumor initiation and its subsequent progression. Given its critical role in oncogenesis and cancer progression, EZH2 is under investigation as a potential biomarker for cancer diagnosis and prognosis. This review provides a comprehensive overview of EZH2’s function and oncogenic roles across human cancers. Enhanced insight into EZH2’s complex regulatory network may facilitate the development of more effective strategies to manage EZH2-driven malignancies.

## Simple Summary

Enhancer of Zeste Homolog 2 (EZH2) is a protein widely recognized for its role in gene silencing and plays a significant role in cancer-related processes, including cell survival, proliferation, invasion, and self-renewal. As the catalytic subunit of Polycomb-Repressive Complex 2 (PRC2), canonically EZH2 mediates the trimethylation of histone H3 at lysine 27 (H3K27), leading to transcriptional repression. Dysregulated EZH2 expression is a hallmark of numerous cancers, both solid and hematologic, and is frequently associated with enhanced metastasis and poor patient prognosis. Notably, EZH2 also exhibits non-canonical functions, including gene activation, which can further promote tumor progression. Due to its significant involvement in oncogenesis and therapy resistance, EZH2 expression is being explored as a diagnostic and/or prognostic biomarker. This review summarizes the function and diverse roles of EZH2 across human cancers, highlighting its potential as both a diagnostic and/or prognostic biomarker. A deeper understanding of EZH2’s intricate regulatory network may enable the development of more effective strategies for managing EZH2-driven malignancies.

## 1. Introduction

Enhancer of zeste homolog 2 (EZH2) is an evolutionarily conserved histone methyltransferase that mediates transcriptional silencing by catalyzing the trimethylation of histone H3 at lysine 27 (H3K27me3) [1,2]. EZH2 functions as the catalytic subunit of the polycomb repressive complex 2 (PRC2), a highly conserved polycomb group protein complex composed of core subunits EZH2, Embryonic Ectoderm Development (EED), Suppressor of Zeste 12 homolog (SUZ12), and Retinoblastoma-binding Protein 7 (RBBP7), along with several accessory factors [3,4]. Together, these components regulate chromatin structure and gene expression [5,6,7]. Within PRC2, EZH2 establishes the H3K27me3 mark, while EED binds both EZH2 and histone H3, acting as a scaffold protein [8]. Structural studies have shown that the EZH2 EED-binding domain (EBD) interacts with the WD repeat domain of EED [9]. SUZ12 is essential for nucleosome recognition, catalytic activity, and overall stability of the complex [10], while accessory proteins such as AE Binding Protein 2 (AEBP2), Polycomb-like (PCLs), and Jumonji AT rich Interactive Domain 2 (JARID2) fine-tune PRC2 activity [9,10]. Once established, the H3K27me3 mark recruits the Polycomb Repressive Complex 1 (PRC1), which mono-ubiquitinates histone H2A at lysine 119, promoting chromatin compaction and stable transcriptional silencing [11]. In addition, EZH2 cooperates with other epigenetic regulators, including DNA methyltransferases (DNMTs) and histone deacetylases (HDACs), suggesting cross-talk among different silencing pathways in the control of gene expression [1,2].

**Figure 1:**
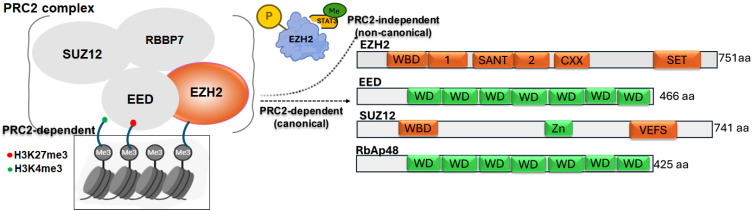
Schematic representation of the PRC2 complex showing core components (EZH2, EED, SUZ12, RBBP7) and their role in catalyzing H3K27me3-mediated gene silencing. A non-canonical function of EZH2 is also depicted, where its phosphorylation enables STAT3 methylation, promoting tumorigenicity. Domain structures of each PRC2 subunit are shown, highlighting key functional motifs involved in complex assembly and enzymatic activity.

Emerging research has identified a non-canonical role for EZH2 as a transcriptional co-activator, potentially mediated through the methylation of non-histone proteins [3,4]. In this PRC2-independent context, EZH2 directly associates with and modifies non-histone targets, leading to activation of downstream gene expression [12]. Through these mechanisms, EZH2 regulates key cancer-related processes, including cell survival, proliferation, invasion, and senescence [13]. Aberrant EZH2 expression is frequently observed across diverse cancer types highlighting its critical contribution to tumorigenesis [14]. Importantly, the functional impact of EZH2 alterations is highly context-dependent, with gain-of-function changes in EZH2 often drive oncogenic activity, whereas loss-of-function alterations confer tumor suppressive effects in certain malignancies [1,2]. This review examines the multifaceted roles of EZH2’s in human cancers, linking its molecular mechanisms to clinical features, pathological outcomes, and patient prognosis. Figure 1 illustrates both the canonical and non-canonical modes of EZH2 action.

## 2. EZH2 Structure

**Figure 2:**
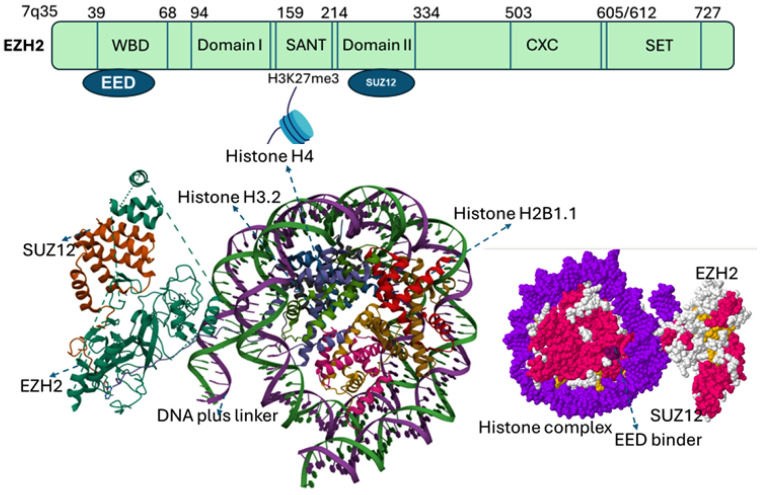
Categorized domains of EZH2. Five functional domains: a C-terminal SET domain, an adjacent cysteine-rich CXC domain, domain I, domain II, and an EED interaction domain (EID)**.** The bottom panel illustrates the PRC2 complex structure with EZH2 (purple), SUZ12 (orange), and EED (gray surface). EED binds to EZH2 to form the core of the Polycomb Repressive Complex 2 (PRC2). The catalytic SET domain of EZH2 mediates H3K27 methylation (blue). EED serves as a regulatory subunit, and binding sites for EED inhibitors (yellow) as well as the EED–EZH2 interaction inhibitor (boxed) are indicated. EED Binders are small molecule or peptide that binds EED, disrupting its interaction with EZH2, while an EED–EZH2 interaction inhibitor specifically blocks the EZH2–EED binding, destabilizing PRC2 and impairing its histone methyltransferase activity.

EZH2 gene, located on chromosome 7q35, comprises 20 exons encoding a protein of 746 amino acids. EZH2 contains five major domains, including the EED-interaction domain (EID), Domain I, Domain II, cysteine-rich domain (CXC domain), and C-terminal SET domain (suppressor of variegation 39, enhancer of zeste and trithorax). Structural and biochemical studies of SET domain in histone methyltransferases have elucidated the molecular basis of histone methylation [4,9], revealing a conserved catalytic Asp–His–Ser (NHS) triad essential for recognizing histone peptide tails and binding S-adenosyl-methionine (SAM), the methyl donor [4,15]. Beyond the SET domain, EZH2 harbors additional functional regions. The CXC (cysteine-rich domain) and an ncRBD (non-coding RNA– and a DNA-binding domain) facilitate interactions with regulatory proteins, while N-terminal domain mediate protein–protein interactions critical for PRC2 assembly and function [16] (Figure 2).

## 3. Molecular Alterations of EZH2 in Cancer

The multifaceted role of EZH2 in cancer has been demonstrated through changes in DNA, posttranslational modifications, and interactions with other epigenetic regulators that collectively modulates its activity. Hyperactivation of EZH2, whether by amplification or mutation, is common in diverse human cancers [17]. A well characterized example is the heterozygous DNA-mediated change at tyrosine 641 (Y641) within the SET domain [18]. Initially thought to be loss-of-function, Y641 alteration (including Y641F, Y641N, Y641S, Y641H, and Y641C) instead confer gain-of-function, shifting substrate preference from unmethylated or monomethylated H3K27 to dimethylated H3K27 (H3K27me2) [17,18,19]. In combination with wild type EZH2, this leads to enhanced accumulation of H3K27me3. Another less frequent amino acid alteration, A677G, also increases catalytic activity on H3K27me2, but unlike Y641 variants, it retains activity toward all three substrates (H3K27, H3K27me1, H3K27me2), reflecting a distinct mechanism [20].

Beyond genomic alterations, posttranslational modifications critically regulate EZH2 activity. Phosphorylation at Ser21 by Akt redirect EZH2 towards non-histone substrates such as androgen receptor (AR), enhancing AR target gene transcription independently of PRC2 [21,22]. Similarly, phosphorylated EZH2 promotes methylation and activation of signal transducer and activator of transcription 3 (STAT3) signaling [12], in part via interaction with SUZ12. Phosphorylation by MELK, a maternal embryonic leucine-zipper kinase, activates NF-κB, driving tumorigenesis and self-renewal [12], while cyclin E/CDK2-mediated phosphorylation at of EZH2 at Thr416 increases EZH2 activity to promote invasion [23]. In contrast, phosphorylation of EZH2 at threonine 311, which is mediated by AMP-activated protein kinase (AMPK), leads to a disruption in the physical interaction between EZH2 and its essential partner SUZ12 [22]. This disruption significantly impairs the histone methyltransferase (HMTase) activity of EZH2. Consequently, this alteration results in the release of the transcriptional silencing that is typically imposed on tumor suppressor genes through the canonical repressive functions of EZH2 [22,23].

EZH2 also engages in crosstalk with other epigenetic regulators. Physical interactions with DNA methyltransferases (DNMT1, DNMT3A, and DNMT3B) recruit DNMTs to EZH2 target loci, linking H3K27me3 with CpG hypermethylation in cancer [24,25]. Similarly, EZH2 transiently interacts with histone deacetylases (HDAC1, HDAC2), which may remove acetyl groups from H3K27 or other lysine residues to facilitate PRC2-mediated methylation [26,27,28,29]. Antagonistic histone marks such as H3K27ac, H3K4me3, and H3K36me2 counteract EZH2 function, highlighting the importance of local chromatin context in determining EZH2 activity [30,31,32]. Collectively, these findings underscore the context-dependent roles of EZH2. Furthermore, upregulation or aberrant activation of EZH2 can silence tumor suppressor genes via canonical PRC2-mediated promoter methylation or act as a non-canonical co-activator of oncogenic pathways [32,33].

**Figure 3:**
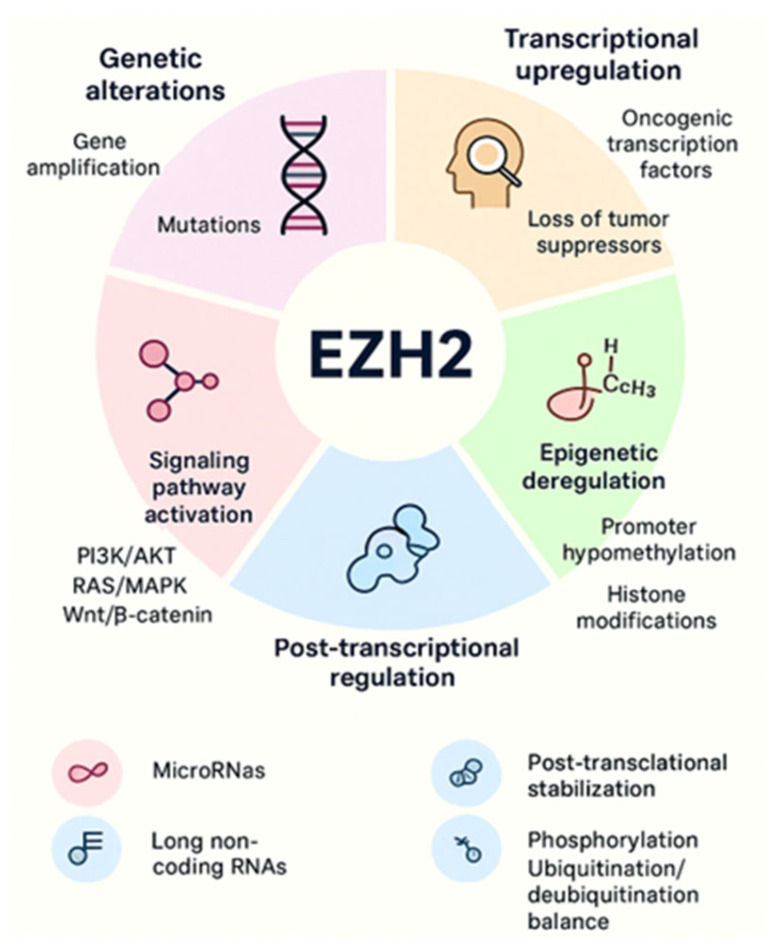
EZH2 overexpression and aberrant activity arise through multiple mechanisms, including genetic alterations, transcriptional upregulation, epigenetic deregulation, signaling pathway activation, post-transcriptional regulation, and post-translational modifications. Together, these processes enhance EZH2 expression and activity, promoting cancer initiation and progression.

Increased EZH2 activity is consistently associated with tumor initiation, progression, and poor prognosis across both solid and hematologic malignancies. Multiple mechanisms of EZH2 regulation, including transcriptional regulation, mRNA regulation by miRNAs, accessibility to DNA via DNA binding proteins and ncRNAs, and post-translational modifications. EZH2 is overexpressed in cancer due to several factors, including transcriptional activation by oncogenic proteins like MYC and ETS family members, deletion, or downregulation of EZH2-inhibiting miRNAs such as miR-101 and miR-26a, and gene amplification in some solid cancers [34,35]. These mechanisms lead to increased EZH2 protein levels, promoting cancer cell proliferation, invasion, and overall tumor aggressiveness. A schematic illustration is shown in Figure 3. More detailed context-dependent studies on the oncogenic roles of EZH2 in cancer have been previously published by our group [1,6,22].

## 4. EZH2 Protein Interactions in Cancer

**Figure 4:**
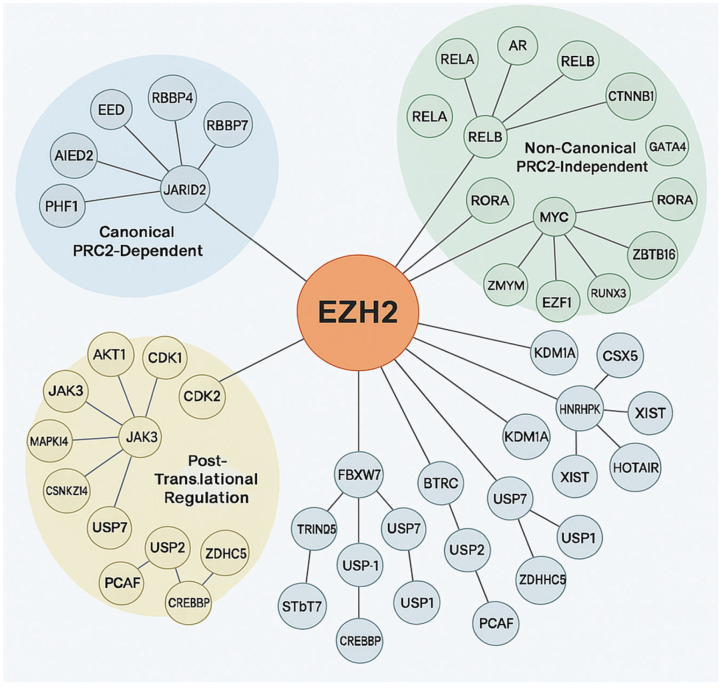
Protein–protein interactions of EZH2. The central orange node represents EZH2, with connecting lines indicating direct or indirect molecular interactions. The surrounding nodes are grouped and color-coded based on functional categories: Canonical PRC2-Dependent (blue cluster). Non-Canonical PRC2-Independent (green cluster): Post-Translational Regulation (yellow cluster), with expected number of edges: 102, PPI enrichment *p*-value: <1.0 × 10^−16^, average node degree: 14.7, avg. local clustering coefficient 0.707.

EZH2 is known for its role in transcriptional repression via H3K27me3 and also participates in PRC2-independent, non-epigenetic pathways by interacting with diverse oncogenic and tumor suppressor proteins [34]. Post-translational modifications tightly regulate EZH2’s enzymatic function, stability, and adaptability in cancer [36]. EZH2 protein–protein interaction network highlights its dual role in epigenetic and non-epigenetic oncogenic processes and its complex involvement in tumorigenesis. This involvement includes canonical PRC2-mediated repression by maintaining cancer cell identity and hindering differentiation through interaction with accessory proteins like JARID2, AE Binding Protein 2 (AEBP2), PHD Finger Protein 1 (PHF1) and PHF19, Metal-Response Element Binding Transcription Factor 2 (MTF2), which are critical for modulating chromatin targeting and enhancing H3K27 methylation [37]. Through non-canonical/post-translational mechanisms EZH2 enables dynamic responses to oncogenic stress, immune evasion, and therapy resistance. EZH2 interacts with oncogenic transcription factors such as AR in prostate cancer, MYC in lymphomas and medulloblastomas, STAT3 in glioblastoma and breast cancer, where it can enhance STAT3 activity through methylation, and binds RelA/RelB thereby potentiating NF-κB signaling and CTNNB1 in Wnt-driven tumors [12]. It also represses or destabilizes tumor suppressors viz. Retinoic acid-related Orphan Receptor Alpha (RORA), GATA4, Runt-related transcription factor 3 (RUNX3), Zinc finger and BTB domain containing 16 (ZBTB16/PLZF) through direct methylation or recruitment to repressive complexes. Interactions with Lysine-specific histone demethylase 1A (KDM1A and/or LSD1) and Heterogeneous Nuclear Ribonucleoprotein K (HNRNPK) involve chromatin and RNA processing, while lncRNA scaffolding including X-inactive-specific transcript (XIST), HOX Transcript Antisense RNA (HOTAIR), Metastasis Associated Lung Adenocarcinoma Transcript 1 (MALAT1) directs EZH2 to specific genomic loci. This non-canonical activity allows EZH2 to act as a transcriptional co-activator or repressor independently of its methyltransferase activity, promoting oncogenic plasticity [38]. Targeting specific components of this protein–protein interaction (PPI) network offers new avenues for precision cancer therapies beyond enzymatic inhibition. Furthermore, EZH2’s function is modulated by phosphorylation such as AKT1 at Ser21 inhibits H3K27me3, CDK1/2 at Thr345/Thr487 regulates chromatin dynamics, Janus kinase 3/mitogen-activated protein kinase 14 (JAK3/MAPK14) in inflammation and stress and ubiquitination and deubiquitination of E3 ligases FBXW7, BTRC, TRIM28 target for degradation; deubiquitinases USP7, USP21, USP1 stabilization as illustrated in Figure 4 [39]. In conclusion, EZH2 is a key regulator of cancer biology, bridging canonical PRC2-mediated repression with diverse non-canonical functions and post-translational modifications. Its dual capacity to act as both a transcriptional repressor and co-activator, independent of methyltransferase activity, underscores its contribution to oncogenic plasticity and immune evasion. EZH2 regulation involves multiple layers, including phosphorylation, ubiquitination, deubiquitination, and interactions with transcription factors, chromatin modifiers, and lncRNAs, reflecting the complexity of its regulatory network. This multifaceted role establishes EZH2 as a clinically significant diagnostic and/or prognostic biomarker, with therapeutic opportunities extending beyond enzymatic inhibition to targeting its specific interactions and regulatory pathways.

## 5. EZH2 Dysregulation in Human Cancers

In normal human tissues, EZH2 expression is generally maintained at low levels, indicative of its specific and tightly controlled roles in cellular homeostasis. For example, studies examining breast epithelium have shown a median percentage of EZH2-positive epithelial cells in normal terminal duct lobular units of approximately 5.88%, with an interquartile range of 1.89–12.46% [40]. This restricted expression suggests that EZH2’s histone methyltransferase activity is only required in a limited population of cells within these normal structures, possibly for maintaining lineage identity or regulating specific developmental programs. Similarly, normal ovarian tissue exhibits negligible EZH2 immunoreactivity, further supporting the notion that EZH2 is not broadly expressed in adulthood or differentiated tissues [41]. This low baseline expression across various normal tissues underscores the importance of maintaining EZH2 activity within a physiological range, preventing aberrant gene silencing or activation that could disrupt normal cellular function and tissue architecture [1,2,3,4]. The tight regulation of EZH2 in normal tissues likely involves intricate mechanisms controlling its transcription, translation, and protein stability, ensuring its potent chromatin-modifying activity is only deployed when and where necessary for proper cellular function and tissue integrity.

In contrast, EZH2 is frequently modified (epigenetically/post-translationally) or genomically altered in a wide array of human cancers [34]. Elevated EZH2 levels have been observed in solid tumors such as prostate, breast, uterine, gastric, and renal cell carcinomas, among others [42]. This aberrant upregulation contributes to oncogenesis by repressing tumor suppressor genes, altering transcriptional programs, and promoting cellular proliferation, invasion, and survival. The dysregulation of EZH2 in cancer often hijacks these normal physiological roles, leading to the acquisition of aggressive cancer hallmarks. In many human cancers, EZH2 upregulation correlates with increased tumor aggressiveness, enhanced metastatic potential, resistance to therapy, and poor clinical outcomes in most of these solid tumors which are described in detail below [43].

### 5.1. EZH2 and Bladder Cancer

Numerous studies have demonstrated the pivotal role of EZH2 upregulation or dysfunction in the pathogenesis of bladder cancer. In recent work by Li et al. serum samples from bladder cancer patients and normal controls revealed significantly elevated EZH2 levels in the patient cohort [44]. These elevated levels were strongly associated with adverse clinical features, including lymph node metastasis, muscle invasion, increased tumor size, and poor overall prognosis. These findings suggest that serum EZH2 could serve as a promising non-invasive biomarker for assessing disease progression and prognosis in bladder cancer. At the molecular level, EZH2 has been shown to contribute to bladder cancer progression through transcriptional repression of tumor suppressor genes. Specifically, EZH2-mediated silencing of E-cadherin, a key epithelial marker has been implicated in enhancing metastatic potential, particularly in superficial transitional cell carcinoma of the bladder [45]. Further supporting EZH2’s role in tumor aggressiveness, Wang et al. identified an enrichment of cancer stem cell subpopulations with high EZH2 expression during bladder cancer recurrence, highlighting its involvement in tumor relapse and/or treatment resistance [46]. Similarly, Chen et al. demonstrated that pharmacological inhibition of EZH2 significantly reduced tumor growth and invasiveness via suppression of the JAK2/STAT3 signaling pathway, further emphasizing its prognostic relevance [47]. In addition to protein-level regulation, upstream non-coding RNAs also influence EZH2 expression [48]. Min et al. reported that long non-coding RNA SNHG1 [49], for instance, has been found to facilitate bladder cancer progression by upregulating EZH2 expression [50]. These findings reveal a broader regulatory network centered on EZH2 and suggest that both EZH2 and its regulatory partners hold promise as prognostic biomarkers. Table 1 summarizes some of the important studies implicating the role of EZH2 in bladder cancer.

### 5.2. EZH2 and Breast Cancer

Several studies have established a strong correlation between EZH2 amplification or dysregulation and the initiation, progression, invasion, and metastasis of breast cancer, particularly in its more advanced stages [66]. Breast cancer is broadly classified into three molecular subtypes: hormone receptor-positive (HR+) breast cancer, characterized by the expression of estrogen (ER) and/or progesterone receptors (PgR); HER2-positive breast cancer, defined by amplification or upregulation of the human epidermal growth factor receptor 2 (HER2); and triple-negative breast cancer (TNBC), which lacks ER, PgR, and HER2 expression [67]. High EZH2 expressions in breast cancer have been consistently associated with unfavorable clinicopathological features, including higher histological grades, ER and PgR negativity, HER2 positivity, and elevated p53 expression [68]. Mechanistically, EZH2 contributes to breast tumorigenesis through both its canonical, PRC2-dependent HMT activity, repressing tumor suppressor genes via H3K27 trimethylation, and non-canonical pathways, where EZH2 functions as a transcriptional activator or co-activator in signaling networks [69]. In ER-positive breast cancer cells, EZH2 has been shown to directly interact with ER and β-catenin to activate transcriptional programs driven by estrogen and Wnt signaling pathways [70]. Conversely, research by Lee et al. demonstrated that in ER-positive contexts, EZH2 cooperates with ER to recruit PRC2 to NF-κB gene promoters, leading to EZH2-mediated H3K27me3 and constitutive repression of NF-κB target genes [71]. In ER-negative breast cancer cells, EZH2 engages in non-canonical activation, forming a complex with RelA and RelB to enhance NF-κB signaling, thereby promoting inflammatory and pro-tumorigenic transcriptional programs [72]. These findings reveal a dual role for EZH2, acting as a transcriptional repressor in ER-positive contexts, and as a transcriptional activator in ER-negative environments via non-canonical mechanisms. Feng et al. demonstrated that EZH2 is localized in the cytoplasm and nucleus of breast cancer cells in a site-specific phosphorylation manner [73]. More advanced HER2-positive clinical-stage breast cancers exhibiting metastatic lymph nodes were found to contain elevated levels of EZH2 compared to less aggressive cancers with low EZH2 levels. pEZH2-S21 localization in the nucleus has shown a correlation with invasive and metastatic lymph node HER2-positive breast cancer, potentially establishing it as an indicator of invasive breast cancer.

Enhanced EZH2 activities and EZH2-induced H3K27me3 regulate signaling pathways such as the Forkhead box (FOX) transcription factor family, which can induce tumor cell proliferation, migration, and bone metastasis, contributing to breast cancer progression [74]. EZH2 also targets downstream genes associated with anticancer effects including FOXO3, CDH1, RKIP, and CDKN1C [74,75]. By repressing these tumor suppressor genes, EZH2 can promote the development of malignant breast cancer. To confirm the oncogenic role of EZH2, it has been either inhibited [63] or knock down to reverse EZH2-conferred induction of breast cancer [64]. A study conducted by Li et al. [76], suppresses EZH2 in conjunction with PARP inhibition led to excessive autophagy and synthetic lethality in triple-negative breast cancer cells. Mao et al. were able to combat the proliferation and invasiveness of triple-negative breast cancer cells after CRISPR-Cas9-mediated EZH2 knockdown [77]. Apart from these observations, the literature contains a plethora of evidence substantiating the role of EZH2 in breast cancer which is summarized in Table 2.

### 5.3. EZH2 and Cervical Cancer

Upregulation of EZH2 in cervical cancer tissues has been consistently associated with advanced disease stage, lymphatic metastasis, deeper tumor infiltration, and reduced overall patient survival [88]. Functionally, EZH2 acts as a primary regulator of cell cycle and an inhibitor of apoptosis, thereby contributing to tumorigenesis and cancer progression. Its upregulation is positively correlated with activation of the Wnt/β-catenin signaling pathway in cervical cancer, which leads to the upregulation of downstream oncogenic effectors such as β-catenin, c-Myc, and Cyclin D1 [89]. These molecules collectively drive uncontrolled cell proliferation and tumor growth. EZH2 also mediates the oncogenic functions of long non-coding RNA SNHG8 in HPV-positive cervical cancers [90]. Through direct interaction, SNHG8 recruits EZH2 to transcriptionally repress RECK (reversion-inducing cysteine-rich protein with kazal motifs), a known tumor suppressor in cervical cancer. This repression promotes cellular proliferation and inhibits apoptosis, enhancing tumor aggressiveness. Furthermore, elevated EZH2 expression in cervical cancer has been linked to hypomethylation of its own promoter region, suggesting an epigenetic feedback mechanism that reinforces its expression [91]. This hypomethylation has been associated with the suppression of senescence-related genes, further contributing to malignant transformation and sustained cancer cell survival. Furthermore, its strong correlation with disease progression and prognosis positions EZH2 as a compelling biomarker in cervical cancer. Table 3 summarizes studies highlighting the oncogenic role of EZH2 in cervical cancer.

### 5.4. EZH2 and Colorectal Cancer

EZH2 upregulation correlates with poor survival in patients in both early and advanced stage tumors with colorectal cancer (CRC) [95]. In CRC tissues, the long non-coding RNA (lncRNA) LINC01116 is upregulated and promotes tumor cell proliferation by recruiting EZH2, which methylates the Tropomyosin 1 (TPM1) promoter, thereby suppressing its translation [96]. Additionally, EZH2 was found to be inversely associated with miR-31 and in sessile serrated adenomas/polyps in premalignant lesions. EZH2 knockdown in colorectal cancer led to increased miR-31 expression [97]. In CRC, EZH2 has been identified as a potential prognostic marker, with elevated expression associated with reduced overall survival. For instance, its association with KDM2B, a cell cycle regulator, has been demonstrated as downregulation of KDM2B reduces EZH2 expression, suppresses PI3K/AKT pathway components, and delays colorectal cancer cell migration [98]. Conversely, EZH2’s combined expression with other polycomb-group (PcG) proteins BMI1 and SUZ12 and their associated histone modification H3K27me3 were correlated with positive patient survival and greater survival for colorectal cancer [99]. EZH2 may also serve as a predictive marker for chemotherapy response and poor 5-year disease-free survival in patients with rectal cancer [100]. These studies highlight EZH2 as both a biomarker and an oncogene in colorectal cancer, as summarized in Table 4.

### 5.5. EZH2 and Esophageal Cancer

EZH2 has emerged as an independent prognostic factor in esophageal cancer, with elevated expression levels significantly correlating with poor disease outcomes [111]. In esophageal squamous cell carcinoma (ESCC), key independent predictors of poor prognosis include high EZH2 expression, advanced histological grade, and distant lymph node metastasis [112]. Notably, EZH2 is consistently elevated at both mRNA and protein levels in esophageal cancer tissues [113,114]. This upregulation contributes to tumor progression by promoting cellular proliferation and metastasis [115]. Mechanistically, EZH2 drives tumorigenesis through its canonical function as a histone methyltransferase. For instance, LINC00114, a long non-coding RNA, has been shown to promote esophageal cancer development by recruiting EZH2 to DLC1 (Rho GTPase Activating Protein) gene promoter, enhancing H3K27me3 and thereby silencing this tumor suppressor gene [116]. In addition, EZH2 regulates epithelial-to-mesenchymal transition (EMT) in ESCC by modulating the expression of miR-200c and key EMT-related genes, ultimately promoting cancer cell migration and invasiveness [117]. These effects are primarily driven by EZH2’s ability to catalyze H3K27 trimethylation at target gene promoters, altering chromatin structure and gene expression. Forced expression of EZH2 in esophageal cancer cells has been shown to significantly elevate global H3K27me3 levels, emphasizing its role in gene silencing and metastasis [112]. Thus, EZH2 serves as a valuable biomarker for predicting ESCC prognosis and metastatic potential. Evaluation of EZH2 expression may thus aid in stratifying patients and tailoring treatment strategies in ESCC. Key studies implicating EZH2 in esophageal cancer are summarized in Table 5.

### 5.6. EZH2 and Gastric Cancer

EZH2 plays a critical role in promoting tumor cell proliferation and advancing gastric cancer by mediating gene promoter methylation [122]. Inhibition of EZH2 in gastric cancer cells has been shown to induce cellular senescence, primarily through the activation of tumor suppressor genes such as p21 and p16 [123]. Moreover, EZH2 expression is influenced by miRNA dynamics, particularly miR-124 [124]. A decrease in miR-124 levels has been associated with elevated EZH2 expression, while overexpression of miR-124 suppresses EZH2 levels, thereby inhibiting cancer progression in gastric cells [125]. Other miRNAs have also been implicated in the regulation of EZH2. For example, miR-26 interacts with the 3’ untranslated region of EZH2 mRNA and, when suppressed during TET-facilitated gastric carcinogenesis, leads to EZH2 upregulation [126]. Additionally, circular RNAs (circRNAs) modulate EZH2 expression in gastric cancer. circKIF4A has been shown to regulate EZH2 via interaction with miR-144-3p. When miR-144-3p is inhibited, the tumor-suppressive effect of circKIF4A is diminished, resulting in increased EZH2 expression [127]. Similarly, circGSK3B facilitates EZH2 upregulation by blocking its binding to the RORA promoter, thereby reducing EZH2 repression [128]. Clinically, EZH2 upregulation in gastric cancer correlates with aggressive tumor phenotypes, including larger tumor size, lymph node metastasis, and lymphatic invasion [128]. Elevated EZH2 levels have also been associated with advanced clinical stages and poor prognosis. One study reported that 68.6% of gastric cancer patients exhibited an increased EZH2 expression [122]. Collectively, these findings underscore the oncogenic role of EZH2 in gastric cancer and highlight its potential as a prognostic biomarker. Key studies illustrating EZH2’s involvement in gastric cancer is summarized in Table 6.

### 5.7. EZH2 and Glioblastoma

Glioblastoma (GBM) is an aggressive brain tumor originating from glial tissue, with a low five-year survival rate of 5.5% and abnormal methylation patterns [140]. EZH2 functions as an oncogene in GBM, contributing to numerous tumor-promoting processes such as cell cycle progression, invasion, glioma stem cell maintenance, resistance to chemotherapy and radiotherapy, angiogenesis, apoptosis inhibition, and tumor proliferation [141]. One key mechanism involves the upregulation of EZH2 leading to increased H3K27 trimethylation, which in turn suppresses the expression of the tumor suppressor PTEN. This suppression activates the PI3K/Akt signaling pathway, promoting enhanced proliferation and migration of GBM cells [142]. Additionally, phosphorylation of EZH2 can lead to increased STAT3 expression through epigenetic methylation, thereby suppressing apoptosis and further advancing GBM progression [12]. EZH2 also cooperates with DNA methyltransferases to regulate miRNA expression, further influencing glioma biology. For example, EZH2 and DNMT1 have been shown to co-mediate the silencing of tumor-suppressive miRNAs, such as miR-200b and miR-429, thereby promoting GBM development [143]. Moreover, higher EZH2 expression facilitates an oncogenic axis by interacting with HP1BP3 and activating WNT7B, a pathway that has been linked to therapeutic resistance [144]. These findings underscore the significant role of EZH2 in the pathogenesis and progression of glioblastoma, supporting its utility as a prognostic biomarker. Additional key studies exploring EZH2’s role in GBM are summarized in Table 7.

### 5.8. EZH2 and Head and Neck Cancer

EZH2 upregulation is correlated with aggressive tumor activity and unfavorable patient survival in head and neck squamous cell carcinoma (HNSCC) [150]. Elevated EZH2 expression in HNSCC is associated with enhanced tumor proliferation and metastatic potential. In particular, silencing EZH2 was shown to upregulate E-cadherin expression, a key epithelial marker, thereby reducing cancer cell migration and invasiveness in HNSCC [150]. Moreover, high EZH2 expression has been linked with lymph node metastasis, a critical prognostic indicator often associated with reduced overall survival in HNSCC patients [151]. However, some contradictory findings have emerged. For instance, the same study reporting EZH2 association with lymph node metastasis did not find a statistically significant relationship between EZH2 expression and patient survival outcomes [150]. Additionally, another study revealed that younger HNSCC patients exhibited lower EZH2 expression levels compared to older counterparts, suggesting that age-specific expression patterns may influence disease behavior and prognosis [152]. This observation implies that EZH2 could serve as a prognostic marker. Overall, while many studies suggest EZH2 is involved in the pathogenesis of HNSCC and could serve as a valuable biomarker or treatment target, conflicting evidence underscores the need for further research. Understanding the context-dependent roles of EZH2 in head and neck cancers will be essential for developing precision therapies. Key studies elucidating EZH2’s role in HNSCC are summarized in Table 8.

### 5.9. EZH2 and Kidney Cancer

Types of kidney cancer include renal cell carcinoma (RCC), transitional cell cancer (TCC), clear cell renal carcinoma (ccRCC), and Wilms tumor. Among these, RCC is the most prevalent, and numerous studies have demonstrated a strong association between EZH2 upregulation and poor clinical outcome [154]. Elevated EZH2 levels have been shown to enhance proliferation and invasion of the RCC cell line ACHN through activation of the Wnt/β-catenin signaling pathway [155]. Additionally, high EZH2 levels represses E-cadherin, a key tumor suppressor gene, and correlates with advanced disease stages and reduced survival in RCC patients [156]. In ccRCC specifically, higher EZH2 level is linked to increased expression of vascular endothelial growth factor, augmented tumor cell proliferation, and reduced apoptosis, aligning with more aggressive clinicopathological features and shorter patient survival [157]. Beyond its pro-proliferative effects, EZH2 can epigenetically silence various tumor suppressor genes and signaling pathways. For example, EZH2-mediated methylation of the Runt-related transcription factor 3 (RUNX3) promoter leads to transcriptional silencing of RUNX3, thereby promoting cancer cell proliferation [158]. Furthermore, high EZH2 expression has been associated with the presence and activation of tumor-infiltrating immune cells, suggesting a broader role in modulating the tumor microenvironment. Moreover, EZH2 depletion results in the re-expression of the cell cycle inhibitor p27/Kip1 and reduced proliferation of RCC cells [159]. EZH2 knockdown has been shown to decrease global levels of histone H3 trimethylation in ACHN cells, reinforcing its role as a key epigenetic regulator in RCC progression. Collectively, these findings underscore EZH2 as a novel prognostic marker in kidney cancer. Additional relevant studies are summarized in Table 9.

### 5.10. EZH2 and Liver Cancer

EZH2 is highly expressed in hepatocellular carcinoma (HCC) and hepatoblastoma tumor tissues and plays a critical role in promoting tumor progression through the regulation of various oncogenic and epigenetic mechanisms [166]. In HCC, one study demonstrated that EZH2 suppresses miR-381 by catalyzing H3K27me3 deposition at its promoter region, thereby enhancing SETDB1 expression and activating the AKT signaling pathway to drive tumorigenesis [167]. Furthermore, EZH2 has been shown to epigenetically silence PD-L1 by increasing H3K27me3 levels at the CD274 and IRF1 promoter regions, undermining immune checkpoint regulation and contributing to immune evasion in HCC [168]. EZH2 is also characterized by a high tumor transformation in liver cancers, and its genomic status has been associated with reduced progression-free and overall survival [169]. Conversely, suppression of EZH2 expression in liver cells leads to the upregulation of tumor suppressor proteins such as p16 and p27, contributing to inhibited tumor growth [170]. Moreover, O-linked N-acetylglucosamine transferase (OGT) expression, which is normally repressed by p53, indirectly promotes miR-15a activity, destabilizing EZH2 and attenuating HCC progression [171]. Similarly, forced expression of miR-101 in HCC cells suppresses EZH2 levels, leading to reduced oncogenic potential [172]. Beyond HCC, EZH2 has also been implicated in cholangiocarcinoma. EZH2 silencing in cholangiocarcinoma cells reduced DNA methylation at the RUNX3 promoter, thereby restoring its tumor-suppressive activity and contributing to decreased liver tumor cell proliferation [173]. These findings emphasize the critical role of EZH2 in liver cancer development and progression, highlighting its promise as a prognostic biomarker. Key supporting studies are summarized in Table 10.

### 5.11. EZH2 and Lung Cancer

EZH2 exhibits oncogenic activity in lung cancer primarily by inhibiting gene transcription via promoter methylation. This epigenetic silencing contributes to tumor cell proliferation and cancer progression. A key pathway involves the immune checkpoint protein programmed death-ligand 1 (PD-L1), whose expression has been shown to correlate positively with EZH2 levels in lung adenocarcinomas [183]. Elevated expression of thyroid transcription factor-1 (TTF-1) a diagnostic marker for metastatic lung tumors-combined with low EZH2 expression, was associated with significantly improved recurrence-free survival in patients [184]. Higher EZH2 expression has also been linked to lung cancers characterized by increased KRAS and BRAF activity, particularly in lung squamous cell carcinoma [185]. Functional studies have demonstrated that silencing EZH2 in parental H2087 lung cancer cells lead to reduced expression of VEGF-A, decreased phosphorylation of AKT at Ser473, and suppression of cell proliferation, migration, and metastasis [186]. In contrast, higher levels of EZH2 in A549 cells promoted these oncogenic traits, suggesting that EZH2 facilitates lung cancer progression via the VEGF-A/AKT signaling pathway [186]. EZH2 is also highly specific to malignant phenotypes. For instance, high EZH2 expression is more frequently observed in malignant mesothelioma, a rare cancer of the pleural lining than in benign proliferative conditions [187]. In non-small cell lung cancer (NSCLC), aberrant EZH2 expression has been associated with poor disease-free survival outcomes [188]. Moreover, its expression is elevated in bronchial preneoplastic lesions, with levels increasing as lesions progress toward malignancy [185]. These findings strongly support EZH2 as a viable prognostic biomarker and therapeutic target in various forms of lung cancer. Further supporting studies are detailed in Table 11.

### 5.12. EZH2 and Nasopharyngeal/Oral Cancer

EZH2 upregulation has been shown to promote the proliferation and migration of endothelial and nasopharyngeal carcinoma (NPC) cells through multiple mechanisms. One pathway involves EZH2-mediated inhibition of miR-1, resulting in increased expression of endothelin-1 (ET-1), a molecule known to promote tumor cell migration and angiogenesis [198]. Additionally, high EZH2 expression has been correlated with p63, a protein involved in epithelial regeneration and associated with significantly lower five-year disease-free survival in patients with NPC [199]. EZH2 has also been implicated in impairing the DNA repair response in NPC. Elevated levels of EZH2 expression were found to suppress the XPA gene, a key component of the nucleotide excision repair pathway [200]. In advanced-stage NPC, this inverse relationship between EZH2 and XPA was evident, and EZH2 inhibition led to increased XPA expression, thereby enhancing DNA repair and accelerating the removal of UV-induced 6-4PP and CPD-DNA adducts [200]. Moreover, EZH2 has been shown to counteract tumor suppressive mechanisms. For instance, miR-506 promotes apoptosis and inhibits proliferation and migration of NPC cells while concurrently downregulating EZH2 [200,201]. In addition, long non-coding RNA H19 has been shown to regulate EZH2 expression by suppressing miR-630, thereby activating the miR-630/EZH2 axis, which enhances NPC cell migration and oncogenic activity [202]. Modulating EZH2, either directly or via regulatory RNAs such as miR-506 or H19, offers a promising strategy for prognostication [203]. These findings highlight the multifaceted role of EZH2 in nasopharyngeal carcinoma as a prognostic marker. Additional supporting studies are summarized in Table 12.

### 5.13. EZH2 and Ovarian Cancer

EZH2 is closely associated with increased malignancy and progression in ovarian cancer, primarily due to its ability to downregulate tumor suppressor genes and repress cell cycle inhibitors, thereby preventing cellular senescence [41]. Specifically, EZH2 has been shown to inhibit the expression of p53, a crucial tumor suppressor gene that normally functions to slow tumor formation, in ovarian cancer tissues [210]. Higher EZH2 expression correlates with therapeutic resistance by promoting DNA replication and cell proliferation [211]. Conversely, EZH2 knockdown results in decreased levels of TGF-β1, a cytokine involved in pathological suppression of normal cellular functions, and an increase in E-cadherin expression, a key component of adherens junctions with tumor-suppressive properties [212]. By inhibiting EZH2, E-cadherin-mediated cellular adhesion and normal cell function are preserved, thereby reducing the proliferation of abnormal ovarian cells. EZH2’s role also extends to the regulation of ferroptosis, a form of programmed cell death recently implicated in ovarian cancer. Upregulation of EZH2 prevents ferroptosis induction, whereas blocking EZH2 expression increases ferroptotic cell death [213]. Furthermore, elevated EZH2 expression is consistently associated with advanced clinical stages of ovarian cancer and is implicated in the progression of diverse subtypes, including ovary granulosa cell tumors [41], small cell carcinoma of the ovary hypercalcemic type (SCCOHT), and high-grade ovarian serous carcinoma (TIL-HGOSC) [214]. Collectively, these findings underscore the significant potential of EZH2 as a prognostic biomarker in ovarian cancer. Additional relevant studies are summarized in Table 13.

### 5.14. EZH2 and Pancreatic Cancer

EZH2 signaling and methylation significantly contribute to the accelerated progression of pancreatic cancer cells. It plays a critical role in regulating cancer cell proliferation, migration, invasion, apoptosis, and cell cycle progression by modulating key signaling pathways such as Wnt, RAS, NF-κB, and NOTCH [222]. Additionally, EZH2 expression induces silencing of E-cadherin via hypermethylation of its promoter, a hallmark associated with metastasis and the development of pancreatic ductal adenocarcinoma (PDAC) [223]. EZH2 also interacts with tumor-suppressive miRNAs, including miR-218 and miR-26a, which are essential for inhibiting tumor proliferation and metastasis. By collaborating with polycomb repressive complexes PRC1 and PRC2, EZH2 promotes methylation of these miRNA promoter regions, silencing their expression in pancreatic cancer [224,225]. Similarly, EZH2 represses tumor suppressor genes like p16INK4, which normally functions to limit tumor proliferation and regeneration, thereby facilitating invasive and metastatic tumor growth [1,2]. Moreover, EZH2 activity is linked to suppression of chemokine signaling and cytotoxic lymphocyte function, correlating with reduced survival in PDAC patients [226]. Given these effects, EZH2 serves as an independent prognostic factor, with higher expression levels predicting poorer clinical outcomes. Combining EZH2 inhibition with senescence-inducing therapies may enhance immune-mediated tumor control in PDAC [227]. Overall, these findings highlight EZH2 as a valuable biomarker for pancreatic cancer prognosis. Additional studies are summarized in Table 14.

### 5.15. EZH2 and Prostate Cancer

EZH2 upregulation is observed throughout most stages of prostate cancer and is strongly associated with aggressive and metastatic disease. Its upregulation promotes oncogenic behaviors largely through the epigenetic silencing of tumor suppressor genes. The androgen receptor (AR), a hormone-activated transcriptional activator critical for prostate-specific cytodifferentiation, plays dual roles: it stimulates prostatic differentiation by promoting transcription of prostate-specific genes while concurrently repressing non-prostatic differentiation through cooperation with EZH2 to inhibit developmental regulators [236,237]. Prostate cancer cell invasion, angiogenesis, and stem cell-like characteristics are linked to EZH2-mediated suppression of interferon-gamma signaling via the PRC2 complex [238]. Beyond its canonical repressive functions, EZH2 also acts as a transcriptional activator or coactivator by binding other transcription factors to promote oncogene expression. For instance, deregulated phosphorylation of EZH2 can switch its function from a PRC2-dependent transcriptional repressor to a coactivator that cooperates with AR, contributing to castration-resistant prostate cancer (CRPC) [237]. Moreover, EZH2 contributes to CRPC through non-canonical mechanisms, such as directly occupying the AR promoter or methylating AR itself, enhancing AR-mediated transcription without the need for other PRC2 subunits [239]. Conversely, EZH2 can suppress AR expression in a PRC2-dependent manner [237]. EZH2 also methylates FOXA1, which recruits deubiquitinases that prevent FOXA1 degradation, elevating its protein levels [240]. Since EZH2 and FOXA1 co-regulate cell cycle progression and prostate cancer growth, their elevated expression correlates with poor prognosis [240]. Additionally, EZH2 affects DNA methylation by directly interacting with DNA methyltransferases, promoting hypermethylation of target genes like GSTP1 and RARB2—epigenetic changes frequently observed in advanced prostate cancer stages [241].

Increased EZH2 expression also facilitates the emergence of more lethal neuroendocrine prostate cancer subtypes, independent of AR signaling, characterized by poorly differentiated small-cell neuroendocrine carcinoma phenotypes [242,243]. Loss of AR and its binding to androgen-response elements following PRC2 complex displacement increases lncRNA-p21 transactivation, which promotes EZH2 release from chromatin [244]. Free EZH2 then switches roles from histone methyltransferase to non-histone methyltransferase, methylating STAT3 to promote neuroendocrine differentiation. Concurrently, EZH2 acts as a co-repressor with N-Myc to drive neuroendocrine differentiation in CRPC cells [245]. Multiple studies have investigated EZH2’s involvement in prostate cancer; a selection of key findings is summarized in Table 15.

### 5.16. EZH2 Expression and Sarcoma

Aberrant EZH2 expression has been associated with poor prognosis, distant metastasis, and tumor necrosis in synovial sarcoma [262]. In pediatric soft tissue sarcoma patients, high EZH2 expression correlated with lymph node involvement and distant metastasis at diagnosis, and those with elevated EZH2 levels showed reduced survival probabilities [263]. Similarly, EZH2 expression was found to be elevated in osteosarcoma tissues and cells. Notably, downregulation of lncRNA-ANCR led to decreased EZH2 levels and increased apoptosis of cancer cells, suggesting a potential regulatory relationship that could inhibit tumor proliferation [264]. Moreover, EZH2 inhibition sensitizes retinoic acid-driven senescence in synovial sarcoma [265]. Overall, these studies highlight the potential of EZH2 as a prognostic biomarker in sarcomas. Additional studies are summarized in Table 16.

### 5.17. EZH2 and Skin Cancer

EZH2 has been implicated in the progression and prognosis of various skin cancers. A study found that higher levels of EZH2 correlated with a BCL2-negative phenotype, which is often observed in advanced disease stages and is associated with shorter event-free survival [269]. In Merkel cell carcinoma (MCC), a type of skin cancer, lower EZH2 expression in primary tumors was linked to improved prognosis and survival compared to moderate or strong EZH2 expression [270]. Additionally, EZH2 dysregulation through somatic activating mutations, copy number amplifications, or transcriptional upregulation has been associated with epigenetic silencing of tumor suppressor genes and melanoma immune responses, negatively affecting patient survival. Knockdown of T antigen in MCC cells reduced EZH2 expression, inducing selective cytotoxicity in virus-positive MCC [271]. In uveal melanoma, forced knockdown of the long non-coding RNA PVT1 suppressed tumor growth and increased apoptosis by regulating EZH2 expression [272]. Furthermore, the combined inhibition of EZH2 and BRAF in melanoma cells-especially those harboring the BRAF V600E mutation and EZH2 demonstrated enhanced therapeutic efficacy, highlighting the potential of this approach in melanoma treatment [273]. Overall, these findings underscore the critical role of EZH2 in skin cancer progression. Additional studies are summarized in Table 17.

### 5.18. EZH2 and Thyroid Cancer

EZH2 upregulation is associated with malignant potential in thyroid cancer, promoting it through transcriptional repression of tumor suppressors and maintenance of cells in a stem-cell-like state [286]. EZH2 has been shown to repress the expression of classic tumor suppressor genes such as CDKN2A and p53 directly and reduces the levels of RAD51, leading to the activation of Raf1/ERK and beta-catenin signaling, leading to thyroid cancer progression [286]. EZH2 can also directly control the differentiation of anaplastic thyroid carcinoma cells by silencing the thyroid-specific transcription factor paired-box gene 8 [287]. Furthermore, EZH2 is important in medullary thyroid cancer by affecting ERK and AKT signaling pathways, as well as controlling genes of the Wnt/beta-catenin [288]. Increased EZH2 expression in papillary thyroid cancer upregulates cellular proliferation and migration by affecting the E2-ERɑ signaling pathway [289]. Beyond this, EZH2 can interact with other pathways to drive gene repression. One example of this is EZH2’s interaction with the HOTAIR (HOXA transcript antisense RNA) pathway, which together encourages an immunosuppressive microenvironment [290]. Because of these traits, EZH2 may be a useful prognostic biomarker for aggressive thyroid cancer. Studies show that certain miRNAs could directly target EZH2 and suppress its expression in thyroid cancer, such as miR-124/506 through decreased H3K27me3 and increased H3K27Ac [291]. Further studies show the inhibition of EZH2 in papillary thyroid cancer downregulates cellular proliferation and migration [289]. EZH2 inhibitors can also favorably modify the immune microenvironment. Additional studies are summarized in Table 18.

### 5.19. EZH2 Expression in Hematological Malignancies

Hematological malignancies include a broad group of blood cancers such as leukemia, lymphoma, and myeloproliferative neoplasms (MPNs). MPNs are rare disorders characterized by the uncontrolled production of abnormal red blood cells, white blood cells, and platelets in the bone marrow. Studies have shown that EZH2 genomic alterations are frequently detected in patients with MPNs and are associated with poor clinical outcomes and early events in leukemogenesis [293,294]. Additionally, EZH2 upregulation is correlated with progression to blast phase MPN, and EZH2 aberration may play a critical role in leukemic transformation in these disorders. These findings underline the importance of EZH2 as a prognostic marker in hematological malignancies. Additional studies are summarized in Table 19.

Genomic alterations in EZH2 result in reduced mRNA expression levels in patients with acute myeloid leukemia (AML), myelodysplastic syndromes (MDS), and myelodysplastic/myeloproliferative neoplasms (MPN) [293]. Studies show that EZH2 expression can induce H3K27me3 trimethylation and confer chronic lymphocytic leukemia (CLL) cells a survival advantage [294]. This occurs through the upregulation of the PI3K/AKT pathway by way of IGF1R and MYC [297]. Therefore, higher EZH2 expression contributes to an increased growth potential of leukemic cells. Furthermore, MDS is characterized by clonal hematopoiesis and impaired differentiation and can develop into AML [294]. One study exploring the mechanism of histone methyltransferase EZH2/EHMT2 during the transformation of MDS into AML showed that NHD13 mice with higher levels of EZH2 transformed into AML. This is because EZH2 catalyzes H3K27me3/H3K9me2 to inhibit the transcription of DLX5, thus promoting the transformation from MDS to AML [298]. Beyond other functions, EZH2 is elevated in most T-cell neoplasms, suggesting that EZH2 could function as an oncogenic protein in T-cell tumorigenesis in adult T-cell leukemia [299]. EZH2 inactivation results in significantly reduced leukemia-initiating cells and enhanced differentiation through the silencing of PRC2 target genes [300]. Furthermore, low EZH2 levels resulted in a decrease in HOX genes and ultimately HOXB7 and HOXA9 knockdown in resistance cells, as shown in Table 20.

EZH2 plays an oncogenic role in lymphoma due to its ability to promote transcriptional repression of target genes [303]. EZH2 upregulation was associated with poor survival outcome, high Ki-67 proliferation rate and p53 mutant patterns caused by tumors [304]. EZH2 presence combined with p53 tumor aberrations causes a poor outcome for MCL patients [305]. Increased EZH2 expression was also correlated with poor overall survival in peripheral T-cell lymphoma (PTCL) patients [305]. EZH2 expression is also higher in aggressive B-cell lymphomas, indicating that it may act as an oncogenic protein in these tumors. EZH2 regulations may differ across various signaling pathways in aggressive B-cell lymphomas, highlighting its potential as a prognostic marker [306]. Alterations in the EZH2 gene may also contribute to its increased expression, since one study found a sizable number of follicular lymphoma patients with an altered EZH2 gene [307]. These findings highlight the importance of investigating specific genomic alterations of EZH2, which may serve as prognostic biomarkers. The results as summarized in Table 21.

## 6. Conclusions and Future Perspectives

The role of EZH2 in human malignancies is well established, with its expression frequently linked to clinical outcomes across diverse cancer types. EZH2 upregulation is commonly observed in prostate, breast, lung, and hematologic cancers, where it correlates with poor prognosis and aggressive disease. Conversely, reduced EZH2 expression has been reported in certain myeloid malignancies, underscoring its context-dependent functions. Dysregulated EZH2 influences both oncogenic and tumor-suppressive pathways, reinforcing its importance as a central regulator in cancer biology.

EZH2 regulation is a multifaceted process that is highly dependent on cellular context and cancer type. Traditionally, EZH2 functions as an oncogene through its role as a transcriptional repressor, silencing tumor suppressor genes via H3K27me3 and thereby driving tumorigenesis. However, studies in hormone-regulated cancers reveal that EZH2 is more versatile, capable of acting as a transcriptional activator independent of PRC2 by targeting non-histone substrates. Its regulation is highly context-dependent and influenced by mechanisms such as transcriptional activation by oncogenic factors (e.g., MYC, ETS family), the loss of inhibitory microRNAs (e.g., miR-101, miR-26a), gene amplification, and interactions with DNA-binding proteins and ncRNAs. Moreover, post-translational modifications, interactions with cofactors, and crosstalk with other epigenetic regulators further diversify their activity (Figure 3). Given this heterogeneity, it is essential to identify the precise EZH2 target genes, whether activated or repressed, in each cancer type. Nonetheless, further studies are needed to define its precise mechanisms and downstream targets, which will be crucial for establishing EZH2 as a reliable, potentially cancer-specific diagnostic or prognostic biomarker.

Gene mapping studies have been crucial in identifying EZH2’s involvement in various cancers by characterizing genomic alterations like overexpression, mutations, and fusions. Such gene-specific mapping would help elucidate the molecular basis of EZH2’s oncogenic or tumor-suppressive roles while also supporting the development of more precise biomarkers tailored to the distinct landscapes of different cancers. However, the interpretation of its prognostic biomarkers remains limited by methodological variability, differences in patient populations, and the absence of standardized longitudinal studies. More consistent approaches and larger cohorts are needed to validate EZH2 as a robust prognostic biomarker. Given the complex regulation of EZH2—shaped by cellular context, cancer type, and specific target genes—defining which genes are activated or repressed by EZH2 in individual malignancies will be essential for tailoring management strategies. At the same time, an important but often overlooked challenge lies in malignancies where loss-of-function EZH2 might contribute to disease progression. Addressing these contrasting roles will be critical for the development of effective EZH2-based biomarkers and precision management strategies in oncology.

## Figures and Tables

**Table 1:** EZH2 expression in bladder cancer.

Year	Authors	N Patients	Method	Clinical Pathological Features/Prognosis
2024	Li et al. [44]	230	ELISA	Elevated EZH2 levels in the serum of patients with bladder cancer are associated with poor prognosis.
2023	Weikert et al. [51]	37	qRT-PCR	Increased EZH2 expression in aggressive and invasive urothelial carcinoma leads to the progression of bladder tumors.
2023	Mohamedali et al. [52]	150	IHC	Strong EZH2 expression and reduced H3K27me3 levels are associated with higher tumor grade, increased proliferative index, and invasive behavior.
2021	Sameh et al. [53]	56	IHC	EZH2 and ARID1A contribute to tumorigenesis and cellular differentiation and may serve as independent prognostic markers.
2021	Zhang et al. [54]	427	qRT-PCR	High EZH2 expression was associated with poor prognosis in bladder cancer patients.
2018	Zhou et al. [55]	189	IHC	EZH2 can serve as a marker for identifying more aggressive phenotypes in patients with urothelial carcinoma.
2018	Chen et al. [47]	34	IHC	EZH2 enhances the proliferation and migration of bladder cancer cells through activation of the JAK2/STAT3 signaling pathway.
2017	Bi et al. [56]	9	WB	Upregulation of EZH2, along with exposure to surgery-induced wound fluid promotes therapeutic resistance in bladder cancer cells.
2016	Joshua Warrick et al. [57]	657	IHC	Correlation of EZH2 status between noninvasive and invasive tumors within individual patients suggests that EZH2 may serve as a marker of tumor lineage.
2016	Chang et al. [58]	375	Statistical Analysis	Aberration in EZH2 gene may be associated with a lower risk of bladder cancer development, particularly in non-smokers.
2016	Yung-Luen et al. [59]	785	qRT-PCR	EZH2 variants may serve as novel susceptibility markers for urothelial cell carcinoma.
2014	Akimasa et al. [60]	171	IHC	Increased EZH2 expression was significantly associated with female gender, ureteral tumor location, sessile architecture, high histological grade, presence of lympho-vascular invasion, concomitant carcinoma in situ, advanced tumor stage, and elevated Ki-67 expression.
2012	Wang et al. [61]	81	IHC	Elevated EZH2 protein expression was associated with more aggressive forms of bladder cancer, including invasive urothelial carcinoma.
2008	Hinz et al. [62]	99	IHC	Members of the PCG family, including BMI1, EZH2, SUZ12, RING1, and CBX7 are expressed in urothelial carcinomas of the bladder.
2007	Hinz et al. [63]	100	qRT-PCR	EZH2 expression was linked to aggressive tumor behavior in urothelial carcinoma and is strongly associated with various pathological features.
2005	Sameh et al. [53]	56	IHC	EZH2 and ARID1A contribute to tumor carcinogenesis and differentiation and may serve as independent prognostic factors in urothelial carcinoma.
2005	Raman et al. [64]	24	IHC	Increased EZH2 expression was correlated with bladder oncogenesis.
2005	Arisan et al. [65]	68	qRT-PCR	EZH2 upregulation precedes increased proliferation rates and the gradual progression of bladder cancer.

**Table 2:** EZH2 expression in breast cancer.

Year	Authors	N Patients	Method	Clinical Pathological Features/Prognosis
2023	Yu et al. [73]	113	IHC	Phosphorylation-dependent EZH2 expression in the cytoplasm and nucleus of breast cancer tissues correlates with lymph node metastasis in HER2-positive cases.
2022	Gan et al. [78]	46	IHC	In ER+ breast cancer, high EZH2 expression correlated with poor prognosis and endocrine therapy resistance, independent of tumor grade and Ki67 status.
2022	Wang et al. [79]	139	IHC	EZH2 inhibition impairs breast cancer progression by suppressing M2 macrophage polarization and infiltration.
2022	Liu et al. [80]	176	RT-PCR	Low EZH2 expression predicted worse survival in TNBC, and EZH2 gene aberration was linked to younger patients (<60 years).
2021	McMullen et al. [81]	35	IHC	pEZH2 T367 patterns vary with metaplastic differentiation and are linked to lymph node metastasis.
2021	Yang et al. [82]	12	WB	EZH2 expression was upregulated post-chemotherapy in patients with poor neoadjuvant response.
2020	Zhou et al. [83]	100	IHC	Positive EZH2 expression was linked to poor prognosis in TNBC.
2019	Dou et al. [84]	48	qRT-PCR	Elevated EZH2 expression in breast cancer tissues was linked to poor prognosis.
2018	Anwar et al. [85]	146	IHC/IF	Cytoplasmic localization and T367 phosphorylation drive EZH2-mediated breast cancer progression.
2018	Boostani et al. [86]	100	IHC	Elevated EZH2 expression was not tied to poor overall survival or disease-free survival but may indicate poor prognosis in breast cancer.
2018	Wu et al. [87]	130	IHC	EZH2 was linked to therapy resistance, with an inverse correlation to GREB1 expression.

**Table 3:** EZH2 expression in cervical cancer.

Year	Authors	N Patients	Method	Clinical Pathological Features/Prognosis
2022	Salmerón-Bárcenas et al. [91]	96	IHC	Increased EZH2 expression in cervical cancer was associated with tumor progression and the suppression of senescence.
2019	Zhang et al. [92]	64	qRT-PCR	HPV18 E6/E7 increases EZH2 and H3K27me3 expression via FOXM1 and E2F-1 binding to the EZH2 promoter and suppress DNMT3A expression.
2017	Azizmohammadi et al. [93]	39	IHC	EZH2 upregulation correlates with the International Federation of Gynecology and Obstetrics (FIGO) stage, histological type, and lymph node metastasis. RIPK4/EZH2 markers may be useful for predicting prognosis in cervical cancer.
2016	Chen et al. [94]	62	IHC	EZH2 expression was 17 times higher in cervical cancer tissues, suggesting its role in cervical carcinoma development and progression.

**Table 4:** EZH2 expression in colorectal cancer.

Year	Authors	N Patients	Method	Clinical Pathological Features/Prognosis
2022	Cheraghi et al. [101]	114	RT-PCR	EZH2 expression was higher in tumor and polyp tissues during colorectal cancer development, indicating its potential as a biomarker.
2021	Abou Gabal et al. [102]	120	IHC	High EZH2 and ERRα expression was linked to shorter overall and progression-free survival, indicating their potential as prognostic markers in CRC.
2021	Sanches et al. [103]	150	IHC	KDM2B downregulation inhibited proliferation, induced DNA damage, and decreased EZH2 expression, activating PI3K/AKT signaling and impairing CRC migration. Their interaction may be a novel prognostic marker for CRC.
2021	Liang et al. [70]	80	IHC	LINC01116 enhances CRC cell proliferation and angiogenesis by recruiting EZH2 to methylate the TPM1 promoter, inhibiting TPM1 transcription.
2016	Kurihara et al. [104]	528	IHC	Elevated EZH2 expression was linked to a favorable prognosis, with an inverse relationship between EZH2 and miR-31 in colorectal cancer.
2016	Chen et al. [105]	81	IHC	High EZH2 expression linked to poorer survival in both early and advanced stage CRC, suggesting it as a predictive marker for prognosis.
2015	Liu et al. [99]	82	qRT-PCR	Elevated expressions of EED, SUZ12, and EZH2 could play a role in the development and progression of CRC.
2015	Lorenzo Fornaro et al. [106]	119	qRT-PCR	EZH2 genotype correlates with higher EZH2 and H3K27me3 immunoreactivity as potential biomarker for EZH2-targeting agents.
2014	Benard et al. [107]	247	IHC, qRT-PCR	Combined expressions of EZH2, BMI1, and SUZ12, along with H3K27me3 modification, provide prognostic value in colorectal cancer.
2014	Meng et al. [100]	112	IHC	Low EZH2 expression in biopsy tissue could predict a better tumor response to neoadjuvant therapy and longer 5-year disease free survival in patients with locally advanced rectal cancer.
2010	Wang et al. [108]	119	IHC	EZH2 and STAT6 expression levels are valuable for distinguishing CRC clinical stages and predicting patient prognosis.
2009	Fluge et al. [109]	412	IHC	EZH2 expression correlated significantly with higher tumor cell proliferation, as measured by Ki-67 expression.
2005	Mimori et al. [110]	61	IHC, qRT-PCR	EZH2 amplification in CRC suggests it as an oncogene and prognostic marker, with concordant expression of HDAC1.

**Table 5:** EZH2 expression in esophageal cancer.

Year	Authors	N Patients	Method	Clinical Pathological Features/Prognosis
2022	Qin et al. [118]	89	qRT-PCR	LINC00114 accelerates EC development by recruiting EZH2, which enhances H3K27me3 on the DLC1 gene.
2020	Qiu et al. [119]	120	qRT-PCR	PSMA3-AS1 is elevated in esophageal squamous cell carcinoma tissues and acts as a miR-101 sponge, thereby upregulating EZH2 expression and contributing to oncogenesis.
2020	Rehman et al. [113]	58	IHC	EZH2 was upregulated in tumors compared to normal tissues, with no link to dysphagia grade with a significant positive correlation with RUNX3 expression.
2020	Zhang et al. [120]	76	qRT-PCR	EZH2 amplification increases ZEB1 expression, with LINC00152 enhancing this effect, promoting epithelial–mesenchymal transition in endometrial cancer cells.
2016	Wang et al. [121]	106	qRT-PCR	MALAT1 downregulation decreases β-catenin, Lin28, and EZH2 expression, while EZH2 upregulation reversed this effect in esophageal cancer cells.

**Table 6:** EZH2 expression in gastric cancer.

Year	Authors	N Patients	Method	Clinical Pathological Features/Prognosis
2024	Ghoreshi et al. [129]	304	qRT-PCR	Reduced miR-124 expression in gastric cancer patients was associated with higher EZH2 mRNA levels, particularly in EBV-infected cases.
2022	Yan et al. [127]	107	qRT-PCR	circKIF4A sponges miR-144-3p to regulate EZH2 in gastric cancer cells, and miR-144-3p inhibition or EZH2 restoration reverses the effects of circKIF4A knockdown.
2021	Ma et al. [128]	56	qRT-PCR	Low circGSK3B and high EZH2 expression correlated with larger tumor size and poor prognosis. circGSK3B inhibited EZH2-mediated RORA suppression, limiting gastric cancer progression.
2020	Li et al. [130]	36	qRT-PCR	Elevated EZH2 expression in gastric cancer counteracts miR-625-3p’s inhibitory effects, promoting tumor progression.
2018	Pan et al. [131]	51	qRT-PCR	Overexpression of miR-124 or inhibition of JAG1/EZH2 reduced fibronectin and vimentin levels in gastric cancer, with miR-124 directly downregulating JAG1 and EZH2.
2018	Gan et al. [122]	156	qRT-PCR	EZH2 was highly expressed in gastric cancer tissues relative to non-tumorous epithelium and correlated with aggressive features and poor outcomes.
2017	Deng et al. [132]	109	qRT-PCR, IHC	TET facilitated gastric cancer by binding miR-26 via its 3′UTR, preventing EZH2 suppression and resulting in EZH2 upregulation.
2016	Sun et al. [133]	85	qRT-PCR	In the cytoplasm, HOXA11-AS acts as a Competing Endogenous RNA (ceRNA) for miR-1297, releasing EZH2 from miR-1297 inhibition and elevating EZH2 expression.
2015	Wang et al. [134]	106	qRT-PCR	XIST knockdown suppressed gastric cancer progression by regulating the miR-101/EZH2 pathway.
2015	Xie et al. [135]	55	IHC, qRT-PCR	HOXA-AS2 knockdown upregulated EZH2-repressed genes, indicating that HOXA-AS2 may inhibit target genes by interacting with EZH2.
2015	Kong et al. [136]	80	IHC, qRT-PCR	PVT1 expression positively correlated with EZH2 protein levels in gastric cancer tissues.
2012	He et al. [137]	117	IHC	Higher EZH2 and H3K27me3 expressions were associated with advanced stages and lymph node metastasis in gastric cancer, but not with age, gender, or tumor grade.
2010	Choi et al. [138]	137	IHC	Elevated EZH2 expression was linked to distant metastases, non-signet ring cell types, and correlated with Ki-67 and p53 levels.
2006	Matsukawa et al. [139]	83	IHC, qRT-PCR	High EZH2 levels correlate with larger tumor size, deeper invasion, and advanced clinical features in cancer.

**Table 7:** EZH2 expression in glioblastoma.

Year	Authors	N Patients	Method	Clinical Pathological Features/Prognosis
2019	Karlowee et al. [145]	12	IHC	High EZH2 expression was linked to shorter overall survival and positively associated with high tumor grade.
2017	Zheng et al. [146]	67	IHC	The positive correlation between EZH2 and NICD1 expression indicates that NOTCH1 could be a potential target of EZH2 in glioblastoma.
2016	Pang et al. [147]	105	IHC	EZH2 expression was inversely correlated with EAF2, suggesting EAF2 as a potential target. Upregulation of EZH2 also activated HIF1α.
2015	Zhang et al. [148]	83	IHC, qRT-PCR	High EZH2 expression correlates with Ki-67 but not with MGMT methylation or IDH1 mutation.
2016	Zakrzewska et al. [149]	53	qRT-PCR	miR-19a, miR-17-5p, and miR-106b expression levels were significantly associated with EZH2 expression.

**Table 8:** EZH2 expression in head and neck cancer.

Year	Authors	N Patients	Method	Clinical Pathological Features/Prognosis
2018	Nienstedt et al. [151]	394	IHC	EZH2 expression was linked to lymph node metastasis but not to tumor grade, stage, surgical margin, distant metastasis, or patient survival.
2016	Chang et al. [153]	90	IHC	High EZH2 expression is linked to advanced T stage, poor survival, and tumor aggressiveness via epithelial-to-mesenchymal transition.

**Table 9:** EZH2 expression in kidney cancer.

Year	Authors	N Patients	Method	Clinical Pathological Features/Prognosis
2022	Lyu et al. [160]	2	RT-qPCR	EZH2 may be a prognostic and microenvironment-associated factor in ccRCC.
2021	Wu et al. [161]	30	qRT-PCR, WB	High EZH2 levels contribute to the overactivation of the IFN-I signaling pathway in systemic lupus erythematosus patients, making EZH2 a promising therapeutic target.
2020	Echenauer et al. [154]	1603	IHC	EZH2 expression and CD8+ cell density are crucial prognostic factors in RCC, with EZH2 upregulation linked to high lymphocyte content.
2018	Sun et al. [162]	62	IHC	Low BRCA1-associated protein 1 (BAP1) levels in ccRCC was linked to poor prognosis and high EZH2 expression.
2017	Ho et al. [163]	1992	IHC	High EZH2 expression in ccRCC doubles mortality risk and improves RCC death prediction, especially in low- and intermediate-risk tumors.
2015	Liu et al. [164]	257	IHC, qRT-PCR	Elevated EZH2 expression correlates significantly with advanced TNM stage.
2016	Karlsson et al. [165]	14	IHC	EZH2 expression was higher in clear cell sarcoma of the kidneys than in Wilms’ tumors, fetal, and adult kidney.

**Table 10:** EZH2 expression in liver cancer.

Year	Authors	N Patients	Method	Clinical Pathological Features/Prognosis
2023	Wu et al. [174]	24	qRT-PCR	High EZH2 expression in HCC patients was linked to poor survival and differed significantly from normal controls.
2022	Zhou et al. [167]	52	qRT-PCR, IHC	EZH2 upregulation in HCC inhibited miR-381 expression via H3K27me3-mediated promoter modification.
2021	You et al. [171]	153	qRT-PCR	OGT, EZH2, and O-GlcNAc were upregulated in HCC tissues, while p53 suppressed HCC development by promoting miR-15a, which destabilized EZH2.
2021	Cui et al. [175]	32	DNA-Seq	Aberration in EZH2 was frequently identified as potential novel biomarkers for liver cancer.
2019	Xiao et al. [168]	386	IHC	Higher EZH2 expression in HCC tumors suppressed PD-L1 in an IFNγ-dependent manner.
2016	Wang et al. [176]	7	WB	EZH2 upregulation in hepatoblastoma drives proliferation by silencing p27.
2015	Zheng et al. [177]	163	IHC, qRT-PCR	Increased EZH2 expression in HCC is tied to poor prognosis, while miR-101 overexpression reduces EZH2 levels in HCC cells.
2014	Gao et al. [178]	151	IHC, qRT-PCR	Increased expression of EZH2 and menin correlates with a poor prognosis in HCC patients.
2014	Xu et al. [179]	99	qRT-PCR	miR-101 expression negatively correlates with EZH2 expression in HCC.
2013	Nakagawa et al. [180]	86	IHC	EZH2 knockdown increased p16 and p27, while upregulation of EZH2 correlated with tumor size in intrahepatic cholangiocarcinoma, lymph node metastasis in extrahepatic cholangiocarcinoma, and Ki-67 in both.
2010	Cai et al. [181]	338	IHC	A positive correlation was observed between H3K27me3 and EZH2 expression in HCCs, with a higher frequency of EZH2 positivity in cases with high H3K27me3 expression.
2009	Yonemitsu et al. [182]	86	IHC	EZH2 expression in HCC was significantly correlated with hypoalbuminemia and advanced TNM stage, whereas BMI1 showed no significant correlation with clinicopathologic factors.

**Table 11:** EZH2 expression in lung cancer.

Year	Authors	N Patients	Method	Clinical Pathological Features/Prognosis
2020	Fan et al. [188]	2180	RNA-seq	High EZH2 expression—alone or synergizing with KRAS/BRAF mutations—predicts poor prognosis in NSCLC, independent of tumor stage or subtype.
2019	Matsubara et al. [189]	350	IHC	Higher EZH2 expression indicates poor NSCLC prognosis.
2018	Toyokawa G. et al. [190]	428	IHC	EZH2 expression in lung adenocarcinomas correlates with increased PD-L1 expression, offering the evidence of their association in resected tumors.
2017	Shinozaki-Ushiku et al. [191]	33	IHC	BAP1 loss and high EZH2 expression are specific markers for malignant mesothelioma, and their combination boosts diagnostic accuracy.
2016	Wang et al. [192]	1695	RNA-seq	Increased EZH2 expression predicts poor prognosis in NSCLC, especially in Asian patients, lung adenocarcinoma, and stage I, but not in Caucasians.
2016	Liu et al. [193]	109	IHC	Higher EZH2 expression predicts poor NSCLC survival and serves as a candidate therapeutic target.
2015	Geng et al. [186]	195	IHC	High EZH2 expression in NSCLC is associated with poor prognosis, larger tumors, higher VEGF-A, and AKT activation.
2014	Xu et al. [194]	360	IHC	EZH2 expression in advanced NSCLC is linked to drug resistance.
2013	Wan et al. [195]	113	IHC	Elevated EZH2 parallels lung cancer development and promotes its progression and metastasis.
2013	Behrens et al. [185]	541	IHC	EZH2 is involved in the initial stages of SCC pathogenesis and correlates with aggressive adenocarcinoma behavior.
2012	Lv et al. [196]	69	IHC, qRT-PCR	EZH2 drives lung adenocarcinoma progression, and its deletion halts cancer growth and restores cisplatin sensitivity.
2012	Huqun et al. [197]	106	IHC	EZH2 promotes NSCLC progression and invasion and serves as a novel prognostic marker.

**Table 12:** EZH2 expression in nasopharyngeal carcinoma/oral cancer.

Year	Authors	N Patients	Method	Clinical Pathological Features/Prognosis
2024	Chen et al. [204]	63	IHC, qRT-PCR	EZH2 boosts cell viability, colony formation, stemness, and epithelial-to-mesenchymal transition in oral squamous carcinoma.
2023	Ganesh et al. [205]	9	IHC	EZH2 expression in oral epithelium predicts oral squamous cell carcinoma transformation in oral leukoplakia and is linked to T-cell infiltration.
2021	Sihavong et al. [206]	78	IHC	EZH2 can indicate disease progression in verrucous lesions and oral verrucous carcinoma and may aid in differentiating oral verrucous hyperplasia from oral verrucous carcinoma in unclear cases.
2020	Sun et al. [207]	86	IHC	In nasopharyngeal carcinoma, EZH2 sustains the stability and inhibitory activity of Tregs induced by EBV-encoded LMP1.
2020	Alajez et al. [201]	15	qRT-PCR	EZH2 upregulation in recurrent nasopharyngeal carcinoma is modulated by miR-26a, miR-101, and miR-98.
2019	Zheng et al. [208]	68	IHC, qRT-PCR	High EZH2 expression correlates with metastasis and poor prognosis in oral squamous cell carcinoma.
2014	Zhao et al. [209]	14	qRT-PCR	EZH2 promotes proliferation, blocks apoptosis, and enhances metastasis and invasion in oral squamous cell carcinoma.
2014	Juan Lu et al. [198]	135	IHC, qRT-PCR	Higher EZH2 expression was associated with increased microvascular density in tumors. EZH2 contributes to angiogenesis in nasopharyngeal carcinoma by downregulating the miR-1/ET-1 axis.

**Table 13:** EZH2 expression in ovarian cancer.

Year	Authors	N Patients	Method	Clinical Pathological Features/Prognosis
2024	Luo et al. [215]	19	RNA-seq	Increased EZH2 expression prevents ferroptosis. Blocking EZH2 may offer potential treatment for ovarian endometriosis.
2023	Chen et al. [216]	105	IHC ChIP-Seq	EZH2 drives ovarian cancer oncogenesis; targeting its noncatalytic activity.
2021	Reid et al. [217]	79	IHC	EZH2 inhibitor–mediated epigenetic reprogramming boosts T-cell and PD-L1–targeted treatments.
2020	Zhai et al. [210]	39	IHC	EZH2 inhibits p53 in ovarian cancer, correlates with stage/grade, and serves as a key diagnostic and prognostic marker.
2020	Sun et al. [218]	63	IHC	EZH2/H3K27me3/pEZH2 predicts chemo response and progression free survival in ovarian cancer.
2020	Huo et al. [219]	160	IHC	EZH2 drives ovarian cancer cell growth and invasion by regulating steroid biosynthesis genes through H3K27me3.
2018	Sun et al. [211]	84	RT-qPCR	Higher EZH2 expression correlates with cisplatin resistance and increased intracellular platinum drug accumulation.
2017	Wang et al. [220]	24	IHC	Inhibiting EZH2 has potential for treatment of small cell carcinoma of the ovary, hypercalcaemic type (SCCOHT).
2016	Xu. et al. [221]	30	IHC	Elevated EZH2 protein level is implicated in ovarian granulosa cell tumor development.

**Table 14:** EZH2 expression in pancreatic cancer.

Year	Authors	N Patients	Method	Clinical Pathological Features/Prognosis
2023	Li. et al. [228]	60	IHC	EZH2 upregulation enhances proliferation and migration in BXP3 cells and could regulate normal pancreatic cell proliferation.
2020	Zhou et al. [229]	42	IHC, qRT-PCR	BLACAT1 interference blocks EZH2 recruitment to CDKN1C, promoting CDKN1C expression, inhibiting CCNE, and suppressing pancreatic cell proliferation.
2018	Ma et al. [230]	105	IHC qRT-PCR	Targeting EZH2 and restoring miR-139-5p could improve prognosis by reducing pancreatic cancer aggression.
2016	Han et al. [231]	84	IHC, qRT-PCR	EZH2 levels were positively associated with clinical stage and lymph node metastasis.
2014	Chen et al. [232]	80	IHC, qRT-PCR	EZH2 expression was positively correlated with Ring1B.
2014	Yamamoto et al. [233]	7	IHC, qRT-PCR	EZH2 knockdown upregulated CEBPA mRNA, but this effect was blocked in KDM6B-KD cells.
2014	Kuroki et al. [234]	181	IHC, qRT-PCR	Increased EZH2 expression in pancreatic IPMN reduces p27Kip1, accelerating cell proliferation in malignant lesions (CIS).
2014	Maftouh et al. [235]	247	IHC, qRT-PCR	EZH2 is a prognostic indicator for advanced/metastatic PDAC, while polymorphisms do not predict clinical outcomes.

**Table 15:** EZH2 expression in prostate cancer.

Year	Authors	N Patients	Method	Clinical Pathological Features/Prognosis
2024	Feschetti et al. [246]	38	IHC, PCR	Blocking AR and EZH2 restrain castration-resistant and neuroendocrine differentiated prostate cancer, re-sensitizes to enzalutamide, and triggers anti-tumor T-cells in prostate cancer.
2023	Zhang et al. [247]	33	IHC/IF	EZH2 controls miR-26a-5p expression in prostate cancer by recruiting H3K27me3 to the promoter.
2022	Su et al. [248]	179	IHC RT-PCR	The circRNA circEZH2E2/E3 suppresses miR363 and miR708 in prostate cancer boosting EZH2 expression via an auto-enhancing loop.
2023	Hansen et al. [249]	90	IHC	TOP2A and EZH2 co-expression could be an independent recurrence predictor.
2021	Huang et al. [250]	30	RT-PCR	SCHLAP1 fosters prostate cancer by using EZH2 to methylate chromosome 5 miRNAs, with DNMT3a feedback.
2019	Ma et al. [251]	42	IHC	EZH2 expression was inversely correlated with FOXO1 protein levels and may negatively regulate FOXO1 in prostate cancer patients.
2019	Xu et al. [252]	120	IHC	EZH2 expression was markedly higher in androgen-dependent and castration-resistant prostate cancer samples. EZH2 staining was more intense in castration-resistant prostate cancer.
2019	Wu et al. [253]	113	IHC	Increased EZH2 expression in prostate cancer biopsies links to higher post-radiotherapy metastasis recurrence.
2018	Bhatia et al. [254]	238	IHC, qRT-PCR	Increased EZH2 expression in SPINK1-positive prostate cancer highlights its role in epigenetically silencing miRNA-338-5p/-421.
2018	Patil et al. [255]	61	IHC	Higher EZH2/SPINK1 protein expression compared to acinar adenocarcinoma underline increased aggressiveness of ductal adenocarcinoma of the prostate.
2018	Lobo et al. [256]	189	IHC	High Ki67, EZH2, and SMYD3 immuno-expression independently predicts outcome in prostate cancer patients at diagnosis.
2017	Labbe et al. [257]	89	IHC	TOP2A and EZH2 are key oncogenic drivers in prostate cancer cells, and EZH2 may identify patients with metastatic potential.
2017	Albdelrahman et al. [258]	70	IHC	Elevated Twist-1 and EZH2, combined with E-cadherin indicate an aggressive prostate tumor with high metastatic risk.
2015	Melling et al. [259]	12427	IHC	EZH2 upregulation in prostate cancer was associated with TMPRSS2:ERG rearrangement, ERG expression, and PTEN loss.
2015	Matsika et al. [260]	142	IHC	ALDH1, EZH2, and SOX2 CSC marker expression varies in prostate adenocarcinomas.
2014	Jacobs et al. [261]	54	IHC	DAB2IP status alongside highest EZH2 could aid in pinpoint high-risk prostate cancer patients with worse prognoses.

**Table 16:** EZH2 expression in sarcoma.

Year	Authors	N Patients	Method	Clinical Pathological Features/Prognosis
2024	Mushtaq et al. [265]	55	NGS	Preferentially Expressed Antigen in Melanoma (PRAME) influences retinoic acid signaling by forming a ternary complex with retinoic acid receptor α (RARα) and EZH2.
2017	Yalcinkaya et al. [266]	29	IHC	EZH2 upregulation indicates poor prognosis in synovial sarcoma and is associated with distant metastasis and necrosis.
2016	Ramaglia et al. [267]	17	IHC	Higher EZH2 expression correlated with lower survival probability and the presence of lymph node and/or distant metastases.
2016	Sun et al. [268]	64	IHC	EZH2 expression is a significant prognostic factor in osteosarcoma, with high expression indicating poor disease-free survival and overall survival, and significantly higher levels observed in non-survivors.

**Table 17:** EZH2 expression in skin cancers.

Year	Authors	N Patients	Method	Clinical Pathological Features/Prognosis
2023	Durand et al. [271]	170	IHC	EZH2 expression was higher in virus-positive than virus-negative Merkel cell carcinoma tumors.
2021	Acikalin et al. [274]	13	IHC	EZH2 plays a key role in Merkel cell carcinoma.
2020	Hoffman et al. [275]	44	IHC	H3K27me3 expression is more common than EZH2 and correlates with a more invasive and metastatic melanoma cell phenotype.
2019	Huang et al. [272]	40	WB	Knockdown of lncRNA plasmacytoma variant translocation gene 1 (PVT1) in uveal melanoma cells inhibits proliferation and promotes apoptosis by regulating EZH2 expression.
2018	Cao et al. [276]	26	IHC	EZH2 plays a relevant role in conjunctival melanoma progression.
2017	Harms et al. [277]	85	IHC	Weak EZH2 expression in the primary tumor (but not nodal metastases) correlated with better prognosis compared to moderate/strong EZH2 expression (5-year Merkel cell carcinoma-specific survival: 68% vs. 22%).
2017	Yu et al. [278]	138	qRT-PCR	BRAF/EZH2 co-inhibition showed superior melanoma prevention suggesting its potential for melanomas with BRAF V600E and EZH2 gain.
2017	Veija et al. [279]	26	NGS	EZH2 upregulation indicates its potential as a drug target in Merkel cell carcinoma.
2016	Tiffen et al. [280]	471	RNA-Seq	Melanoma patients show EZH2 dysregulation, which worsens survival. EZH2 hyperactivation causes DNA methylation and epigenetic silencing of key genes.
2016	Rao et al. [281]	59	IHC	EZH2 expression correlates with the proliferation marker Ki67 and aggressive basal cell carcinoma subtypes, consistent with higher EZH2 expression.
2016	Montagnani et al. [282]	10	Exome-Seq	Higher EZH2 mRNA in metastatic melanoma samples, points to EZH2 as a potential driver of metastasis.
2016	Emadali et al. [283]	47	qRT-PCR, IHC	Glucocorticoid Receptor (NR3C1) deletion in Blastic Plasmacytoid Dendritic Cell Neoplasm (BPDCN) leads to a major downstream effect with loss of EZH2 function.
2016	Harms et al. [284]	15	NGS IHC, qPCR	The gain of ATK1 and EZH2 was observed in 33.3% of patients.
2016	Stacchiotti et al. [285]	263	IHC, qRT-PCR	Fibrosarcomatous Dermatofibrosarcoma Protuberans showed more EZH2-positive nuclei, and a higher Ki-67 score than Dermatofibrosarcoma Protuberans.

**Table 18:** EZH2 expression in thyroid cancer.

Year	Author	N Patients	Method	Clinical Pathological Features/Prognosis
2021	Sawicka-Gutaj et al. [288]	30	qRT-PCR	EZH2 upregulation was potentially linked to papillary thyroid cancer.
2021	Ma et al. [292]	50	qRT-PCR	Coiled-Coil Domain Containing 26 (CCD26) contributes to thyroid cancer’s malignant progression via the miR-422a/EZH2/Sirt6 axis, highlighting EZH2 and Sirt6 as crucial factors in its development.
2019	Xue et al. [289]	65	IHC qRT-PCR	EZH2 plays a role in papillary thyroid carcinoma growth and metastasis.
2018	Masudo et al. [286]	48	IHC	Higher EZH2 expression correlates with malignancy in thyroid cancer and may serve as a prognostic marker for aggressive forms.

**Table 19:** EZH2 expression in myeloproliferative neoplasm.

Year	Author	N Patients	Method	Clinical Pathological Features Prognosis
2018	Lasho et al. [295]	64	NGS	EZH2 upregulation during the blast phase may contribute to leukemic transformation in myeloproliferative neoplasms.
2018	Venton et al. [296]	56	NGS	EZH2 remains frequently altered gene and plays a role in Myeloproliferative Neoplasms transformation into secondary Acute Myeloid Leukemia.

**Table 20:** EZH2 expression in leukemia.

Year	Author	N Patients	Method	Clinical Pathological Features/Prognosis
2020	Stasik et al. [301]	1604	IHC	In leukemia patients, high EZH2 expression was associated with significantly poorer disease-free survival (DFS) and overall survival (OS), with non-survivors showing markedly higher EZH2 levels than survivors.
2016	Shen et al. [302]	50	qRT-PCR	EZH2 was associated with synovial sarcoma, and its knockdown inhibited cell growth and migration across multiple synovial sarcoma cell lines.

**Table 21:** EZH2 expression in lymphoma.

Year	Author	N Patients	Method	Clinical Pathological Features/Prognosis
2024	Kim et al. [304]	81	IHC	EZH2 upregulation in Mantle Cell Lymphoma (MCL) correlates with aggressive histology, high Ki-67, and p53 mutation. Patients with either EZH2 expression or TP53 mutation have worse outcomes, which are dismal when both are present.
2023	Grob et al. [308]	50	qRT-PCR	High EZH2 expression correlated with poorer overall survival rates in nodal MCL.
2021	Schumann et al. [309]	33	IHC	EZH2 and H3K27me3 are upregulated in T-cell lymphomas, and their high expression levels correlate with poorer overall survival and progression-free survival.
2021	Martinez-Baquero et al. [310]	150	IHC	EZH2 expression in Mantle Cell Lymphoma is associated with high proliferation, higher p53 levels, aggressive histology, and worse overall survival.
2022	Wu et al. [311]	41	IHC	EZH2 likely plays a role in MCL pathogenesis and could be a biomarker for predicting clinical outcomes. High EZH2 expression correlates with poor overall survival.
2019	Zhang et al. [312]	82	IHC	peripheral T-cell lymphoma (PTCL) patients show high EZH2 expression, which is linked to worse survival. EZH2 and HDAC2 could serve as prognostic markers in PTCL, particularly PTCL-not otherwise specified.
2019	Deng et al. [313]	136	IHC	In diffuse large B-cell lymphoma (DLBCL), high EZH2 expression strongly associates B symptoms and relapse. EZH2 (H3K27 methyltransferase) and Bcl-2 could be candidate biomarkers.
2017	Huet et al. [314]	159	IHC	EZH2 gene alteration strongly correlated with increased EZH2 mRNA expression.
2016	Wang et al. [315]	40	qRT-PCR	MCL tissues showed significantly elevated EZH2 expression compared to healthy donors. EZH2 levels were also significantly higher in intermediate and high-risk MCL than in the low-risk group.
2016	Oh et al. [316]	231	IHC	High EZH2 protein expression in DLBCL, and HOTAIR expression correlates with EZH2 expression.
2016	Tian et al. [317]	106	IHC	EZH2 upregulation in aggressive B-cell lymphomas indicates its oncogenic function with likely regulation by diverse signaling in different subtypes.

## Data Availability

No new data were created or analyzed in this study.

## Abbreviations

AEBP2: AE binding protein 2; AML, acute myeloid leukemia; AMPK, AMP-activated protein kinase; AR, androgen receptor; BLACAT1, BLACAT1 overlapping LEMD1 locus; BTRC, beta-transducin repeat containing E3 ubiquitin protein ligase; ccRCC, clear cell renal carcinoma; CDKN1C, cyclin-dependent kinase inhibitor 1C; circRNAs, circular RNAs; CLL, chronic lymphocytic leukemia; CRPC, castration-resistant prostate cancer; CXC domain, cysteine-rich domain; DFS, disease free survival; DNMTs, DNA methyltransferases; EBD, EED-binding domain of EZH2; EED, embryonic ectoderm development; EID, EED-interaction domain; ELISA, enzyme-linked immunosorbent assay; ER, estrogen receptor; ESCC, esophageal squamous cell carcinoma; EZH2, enhancer of zeste homolog 2; FBXW7, F-box/WD repeat-containing protein 7; FOX, Forkhead box; GBM, glioblastoma; GSTP1, glutathione S-transferase pi 1; H3K27me3, trimethylation of histone H3 at lysine 27; HCC, hepatocellular carcinoma; HDACs, histone deacetylases; HER2, human epidermal growth factor receptor 2; HMT, histone methyltransferase; HOTAIR, HOX transcript antisense RNA; IGF1R, insulin-like growth factor 1 receptor; IHC, immunohistochemistry; JARID2, jumonji, AT-rich interactive domain 2; KDM1A/LSD1, lysine-specific demethylase 1A; lncRNA, long non-coding RNA; MALAT1, metastasis associated lung adenocarcinoma transcript 1; MCC, Merkel cell carcinoma; MDS, myelodysplastic syndrome; MELK, maternal embryonic leucine-zipper kinase; MPN, myelodysplastic/myeloproliferative neoplasms; MPN, myeloproliferative neoplasm; MTF2, metal response element binding transcription factor 2; NGS, next gen sequencing; NPC, nasopharyngeal carcinoma; NSCLC, non-small cell lung cancer; OS, overall survival; PDAC, pancreatic ductal adenocarcinoma; PD-L1, programmed death-ligand 1; PgR, progesterone receptor; PHF1, PHD finger protein 1; PRC2, polycomb-repressive complex 2; qRT-PCR, quantitative reverse transcriptase PCR; RARα, retinoic acid receptor α; RBBP7, RB binding protein 7; RCC, renal cell carcinoma; RECK, reversion-inducing cysteine-rich protein with kazal motifs; RNA, ribonucleic acid; RORA, RAR-related orphan receptor alpha; RUNX3, RUNX family transcription factor 3; SAM, S-adenosyl-methionine; SCCOHT, small cell carcinoma of the ovary hypercalcemic type; SET domain, enhancer of zeste and trithorax domain; STAT3, signal transducer and activator of transcription 3; TCC, transitional cell cancer; TGF, transforming growth factor; TNBC, triple-negative breast cancer; TRIM28, tripartite motif containing 28; TTF-1, thyroid transcription factor-1; USP, ubiquitin-specific-processing protease; VEGF, vascular endothelial growth factor; WB, Western blotting; XIST, X inactive specific transcript; ZBTB16/PLZF, zinc finger and BTB domain containing 16/promyelocytic leukemia zinc finger.
